# Supplementary material for: Ultrathin silica-tiling on living cells for chemobiotic catalysis
Source: Nat Commun. 2024 Jul 10;15:5773. doi: 10.1038/s41467-024-50255-7 (PMC11233561; doi:10.1038/s41467-024-50255-7)
Supplement: Supplementary file 1 — Supplementary Information [file 41467_2024_50255_MOESM1_ESM.pdf]

Supplementary Information

Ultrathin Silica-Tiling on Living Cells for Chemobiotic Catalysis

Jeongsang Oh,[1,2,5] Nitee Kumari,[1,2,5] Dayeong Kim,[1,2] Amit Kumar,*[1,2,4] In Su Lee*[1,2,3,4]

[1]Creative Research Initiative Center for Nanospace-confined Chemical Reactions (NCCR), Pohang University of Science and Technology (POSTECH), Pohang 37673 (Korea)

[2]Department of Chemistry, Pohang University of Science and Technology (POSTECH), Pohang 37673 (Korea)

[3]Institute for Convergence Research and Education in Advanced Technology (I-CREATE), Yonsei University, Seoul 03722 (Korea)

[*]Correspondence: amitkumar@postech.ac.kr (A.K.); insulee97@postech.ac.kr (I. S. L.)
[4]These authors jointly supervised this work.
[5]These authors contributed equally.

Supplementary Methods

1. General Information

1.1 Instruments and Characterization. Transmission electron microscopy (TEM) and high-resolution TEM (HRTEM) were conducted using JEOL JEM-2100, BIO TEM JEM-1011, BIO TEM Talos L120C. Atomic scale HAADF-STEM analysis was performed using a 200 kV operated scanning/transmission electron microscope (S/TEM) (JEOL ARM 200F) with a spherical aberration corrector (ASCOR, CEOS GmbH, Germany). For HAADF imaging, the camera length was set to 10 cm with inner and outer detector angles ranging between 45-180 mrad and a probe size of 9C. Scanning transmission electron microscope and energy-dispersive X-ray spectrometry (STEMEDS) elemental mapping and line profiling were carried out using JEOL JEM-2100F at 200 kV. Scanning electron microscopy (SEM) was conducted using the HITACHI S-4800 instrument. X-ray photoelectron spectroscopy (XPS) was performed using a K–ALPHA$^+$ XPS system (Thermo Fisher Scientific, UK) equipped with monochromatic Al Kα (1486.6 eV) radiation and the peak was fitted using Avantage software. Powder X-ray diffraction (XRD) patterns were recorded using a D/MAX-2500/PC (18 kW) (Rigaku) diffractometer with Cu-Kα radiation (λ = 0.15418 nm) at 40 kV and 100 mA. UV−vis spectroscopy was carried out with a JASCO V-650 UV−vis spectrophotometer. Each chemical structure and surface charge of the synthesized samples were investigated by Fourier transform infrared spectroscopy (FT-IR, Two IR spectrometer, PerkinElmer) and Zeta-potential measurement (Malvern Instruments, Zetasizer Nano ZS), respectively. The Raman spectra were recorded using a WITECH Alpha 300R Raman spectroscope equipped with a Nd:YAG laser (excitation wavelength: 532 nm). The metal contents were determined by inductively coupled plasma atomic emission spectrometry (ICPAES) using iCAP 7400 (Thermo ScientificTM). Flow cytometry analysis was carried out on a Beckman Coulter (CytoFLEX) instrument and analyzed with CytExpert software. Confocal laser scanning microscopy (CLSM) studies were performed using the broadband confocal Leica TCS SP5 microscope and analyzed with Leica Application Soite software. Analytical thin-layer chromatography (TLC) was carried out on silica gel 60Å F254 plates (Merck KGaA, Pvt. Ltd.) with detection by a UV detector. ^{1}H NMR spectra were recorded on a Bruker Avance 300 (75 MHz); chemical shifts are indicated in parts per million downfield from $SiMe_4$ and analyzed with MestReNova software. The yield calculation was observed by the 6500 GC system (YOUNG IN Chromass) (Column: CPF502, CP-Chirasil(Dex C8), Length 25 m, Diameter 0.25 mm, film 0.25 μm). All graph data were created using Origin 2018 software. The molecular formula was drawn using ChemBioDraw Ultra 14.0 software.

1.2 Reagents and materials. All chemicals, including nickel(II) nitrate hexahydrate (Strem), cobalt(II) nitrate hexahydrate (Aldrich), hexamethylenetetramine (HMT, Acros), sodium dodecyl sulfate (SDS, Acros), formamide (Samchun Chem.), IGEPAL CO-520 (polyoxyethylene (5) nonylphenylether,

Aldrich), IGEPAL CO-720 (Polyoxyethylene (12) nonylphenyl ether, Aldrich), aqueous ammonia (Samchun Chem., 28.0~30.0%), N-[3-(triethoxysilyl)propyl]ethylenediamine (TESD, TCI, 96%) tetraethyl orthosilicate (TEOS, Acros, 98%), N-[3-(trimethoxysilyl)propyl]ethylenediamine (TMSD, Aldrich, 97%), , hydrochloric acid (Samchun Chem., 35.0~37.0%), hydrogen tetrachloroaurate(III) hydrate (Strem), sodium tetrachloropalladate(II) trihydrate (Strem), sodium borohydride (Samchun), sodium tetrachloroplatinate(II) hydrate (Strem), sodium tetrachloropalladate(II) trihydrate (Strem), L(+)-ascorbic acid (Samchun Chem.), trimesic acid (Aldrich), iron(III) chloride anhydrous (Acros), 5 nm gold nanoparticels (Aldrich), lipase B (*Candida antarctica*) immobilized on Immobead 150, recombinant from Aspergillus oryzae (CALB, Aldrich), yeast from Saccharomyces cerevisiae (Aldrich), YPD agar (Aldrich), YPD Broth (Aldrich), Phosphate-Buffered Saline (PBS, Lonza), 2-propanol (Alfa), Methyl Benzoylformate (Acros), Triethanolamine (Acros), beta-nicotinamide adenine dinucleotide (NAD^+, Alfa), beta-nicotinamide adenine dinucleotide reduced disodium salt (NADH, Alfa), Hydrogen peroxide (H_2O_2, SAMCHUN), Lyticase from Arthrobacter Iuteus (Aldrich), Trisodium citrate dehydrate (Kanto chemical), Tannic acid (Aldrich), Silver nitrate (Samchun), Hexadecyltrimethlyammonium bromide (CTAB, Acros, 99%) were directly used as purchased without further purification. TLC was performed on aluminum-backed silica plates, and UV light was used to visualize products. ^1H NMR spectra were recorded using $CDCl_3$ as the solvent, and chemical shifts are reported in ppm downfield from tetramethylsilane. Throughout the experiment, deionized (DI) water was used unless mentioned specifically.

2. Experimental Section

2.1 Synthesis of 2D-SiNTs

Step 1. Synthesis of single-layered (1 nm thick) Nickel Cobalt layered double hydroxide (SL-LDH):

The preparation of the SL-LDH aqueous suspension was carried out using a reported protocol with modification.[1] The DS-LDH, intercalated with dodecyl sulfate (DS), was synthesized by precipitating aqueous solutions of nickel, cobalt, and sodium dodecyl sulfate (SDS) via hexamethylenetetramine (HMT) hydrolysis. Three aqueous solutions were prepared, including a 0.32 M mixture of metal nitrate hexahydrate with a molar ratio of Ni:Co of 3:1, 0.58 M SDS, and 0.36 M HMT solution. These solutions were then mixed with M^{2+} (10 mL), SDS (10 mL), and HMT (20 mL) solutions. The resulting mixture was transferred to a Teflon-lined stainless steel autoclave with deionized water (DI, 40 mL). Subsequently, the solution was heated in a preheated oven at 110 ℃ for 24 h under sealed conditions. After the reaction, the precipitate was collected by centrifugation, washed repeatedly with DI and ethanol, and dried in air at 60 °C. The product was mixed with formamide (1 mg mL^{-1}) to exfoliate the host layer, and the resulting suspension was heated at 40 °C for 24 h without stirring. The suspension

was then centrifuged at 20000 rpm, and the supernatant was washed thoroughly with ethanol and DI, finally to store in dispersed form.

Step 2. Silica encapsulation of SL-LDH:

For silica encapsulation of SL-LDH, we followed a previously reported reverse microemulsion procedure.[2] IGEPAL CO-520 (0.2 mL) was dispersed in cyclohexane (6.6 mL) in a 20 mL glass vial and stirred vigorously using a magnetic stirrer for 10 minutes at room temperature. An aqueous suspension of SL-LDH (0.4 mL, 12.5 mg mL^{-1}) was added dropwise to the solution with continuous stirring to create a homogeneous reverse microemulsion system. After 10 minutes, an aqueous ammonia solution (28~30%, 33.4 μL) and TEOS/TMSD 2% (30 μL) were added dropwise to the suspension at 10 minute intervals to initiate the silica sol–gel reaction. The reaction was kept at room temperature for 24 hours. Additional TEOS (10 μL) was added dropwise to the suspension, and the reaction was kept at room temperature for another 24 hours. The internally amine-functionalized LDH@SiO_2 nanosheets were collected by centrifugation and washed with ethanol and deionized water for further use.

Step 3. Etching of LDH:

Internally amine-functionalized hollow 2D silica bilayer-sheets with a 1 nm nano-gap were synthesized through the acid treatment of LDH@SiO_2 (5 mg) using 3 M HCl (5 mL) under stirring overnight at room temperature. After the reaction, precipitates were obtained by centrifugation and washed with ethanol and deionized water (DI).

2.2 Synthesis of thick (rigid) bilayer silica nanosheets

Step 1. Synthesis 7 nm thick Nickel Cobalt LDH:

A mixture of 0.32 M metal nitrate hexahydrate (Ni:Co molar ratio 3:1) and 7.2 M sodium hydroxide in 10 mL DI is stirred at 600 rpm for 11 min. The suspension is then centrifuged at 12000 rpm and washed thoroughly with DI. The resulting mixture was transferred to a Teflon-lined stainless steel autoclave and DI (40 mL) was added. The solution was then sealed and heated in a preheated oven at 150 °C for 12 h. After the reaction, the precipitate was collected by centrifugation and washed repeatedly with deionized water and ethanol.

Step 2. Silica encapsulation of LDH:

IGEPAL CO-520 (0.2 mL) was dispersed in cyclohexane (6.6 mL) in a 20 mL glass vial and stirred vigorously using a magnetic stirrer for 10 min at room temperature. An aqueous suspension of 7 nm-LDH (0.4 mL, 12.5 mg mL^{-1}) was added dropwise to the solution with continuous stirring to create a homogeneous reverse microemulsion system. After 10 minutes, an aqueous ammonia solution (28~30%, 33.4 μL) and TEOS (40 μL) were added dropwise to the suspension at 10 minute intervals to initiate the silica sol–gel reaction. The reaction was kept at room temperature for 24 hours. The 7nm-

$LDH@SiO_2$ nanosheets were collected by centrifugation and washed with ethanol and deionized water for further use.

Step 3. Etching of LDH

2D silica bilayer-sheets with a 7 nm gap were synthesized through the acid treatment of $LDH@SiO_2$ (5 mg) using 3 M HCl (5 mL) under stirring overnight at room temperature. After the reaction, precipitates were obtained by centrifugation and washed with ethanol and deionized water (DI).

2.3 Synthesis of spherical core@shell silica nanoparticles

A cyclohexane suspension of oleate-capped MnO nanoparticles (12 mg) was injected under vigorous magnetic stirring (800 rpm) into a solution of IGEPAL CO-520 (1.8 mL) in cyclohexane (10 mL). After 10 min, when the suspension became clear, an ammonia solution (28-30%, 0. 2 mL) was added to the reaction mixture. Then, a mixture of TEOS (0.2 mL) and TESD (0.2 mL) was added dropwise first, followed by five successive additions of TMSD (0.01 mL) to the suspension at 10 min intervals. After 24 hours, TEOS (1 mL) was further added to the suspension and stirred continuously for 48 h at room temperature. $MnO@SiO_2$ nanoparticles were collected by centrifugation (14000 rpm. 10 min), washed with ethanol (2x) and deionized water (1x), and dispersed in DI water for further use.

2.4 Synthesis of rod-shaped core@shell silica nanoparticles

0.1M Iron(III) chloride in 50 mL DI was heated in an oven at 40 °C for 30 h, then left at 4 °C for 1 day to prepare nanorod-shaped FeOOH NPs. For silica encapsulation, Stober reaction was initiated by adding 1 mg of FeOOH in 5 mL ethanol and an aqueous solution of ammonia (28-30%, 50 μL) and TEOS (20 μL) dropwise to the suspension at 10 min intervals. The reaction was maintained at room temperature for 24 h. $FeOOH@SiO_2$ was collected via centrifugation and washed with ethanol and DI and stored for further use.

2.5 Mesoporous silica NPs (mSiO2) synthesis.

$mSiO_2$s were synthesized following previously reported methods in our group with no modification.[3] Briefly, IGEPAL 520 (1.2 mL) and IGEPAL 720 (1.2 mL) in cyclohexane (20 mL) under vigorous magnetic stirring, and the resulting suspension was successively treated with aqueous ammonia (28–30%, 200 μL) and DI (25 μL). Then the mixture of TEOS (4.51 M, 100 μL) and TESD (3.56 M, 150 μL) was added two times first, followed by the addition of TMSD (4.62 M, 20 μL) five times into the suspension at every half-hour interval, consecutively. After 24 h, TEOS (4.51 M, 100 μL) was added to the suspension, followed by 48 h of stirring at room temperature. The resulting white colored $mSiO_2$

was collected by centrifugation, washed with ethanol (three times) and deionized (DI) water (one time), and dispersed in DI water for further use.

2.6 Synthesis of Silver Nanoparticles.

Silver Nanoparticles were synthesized by referring to previously reported papers.[4] A 100 mL volume of aqueous solution containing sodium citrate (5 mM) and tannic acid (0.1mM) was prepared and heated with a heating mantle in a three-neck round-bottomed flask for 15 min under vigorous stirring. A condenser was used to prevent the evaporation of the solvent. After boiling had commenced, 1 mL of $AgNO_3$ (25 mM) was injected into this solution. The solution became bright yellow immediately. Resultant Ag NPs were purified by centrifugation (20000*g*) in order to remove the excess of TA and further redispersed in Milli-Q-water.

2.7 Synthesis of AuPt/2D-SiNTs (0.8, 0.4, 0.2) with different amounts of Pt

Initially, 0.5 mL of an aqueous suspension of pre-synthesized negatively charged 2D-SiNTs (2 mg mL^{-1}) was combined with 0.5 mL of a freshly prepared gold precursor solution ($HAuCl_4$•xH_2O, 15 mM). The reaction mixture was stirred for 2 h at room temperature. Subsequently, the mixture was washed twice with DI. Next, 0.2 mL of a sodium borohydride solution ($NaBH_4$, 100 mM) was added to the above solution. The mixture was stirred for 10 minutes at room temperature and thoroughly washed with ethanol and DI to result 2D-SiNTs modified by Au-seeds at the hollow interior. 0.5 mL of 2D-SiNTs containing Au-seeds (2 mg mL^{-1}) was combined with 0.5 mL of a freshly prepared platinum precursor solution (Na_2PtCl_4•xH_2O, (2, 4, 8) mM). The reaction mixture was stirred for 30 min at room temperature. Subsequently, 0.5 mL of an ascorbic acid solution (AA, (2, 4, 8) mM) was quickly added to the above solution. The entire reaction mixture was then kept on a preheated oil bath (70 ℃) under stirring for 10 minutes. Finally, AuPt/2D-SiNTs as the resulting black-colored material was isolated from the reaction solution via centrifugation, washed with ethanol and DI.

2.8 Cell culture and harvesting

Yeast (*Saccharomyces Cerevisiae*) was purchased from Sigma-Aldrich, stored as a powder at 2 °C. Yeast from the powder was cultured overnight at 30 °C on a YPD agar stock plate that was never kept longer than one month. For culturing, a single colony of yeast cells was picked from the agar stock plate and grown in YPD broth (0.2 g yeast extract, 0.4 g glucose, and 0.4 g peptone in 20 mL H_2O) in a shaking incubator at 30 °C for 24 h. Yeast cells were harvested by centrifugation of a 5 mL culture at 2000 rpm for 3 min and washed twice with PBS at pH 7.0 (0.4 mol L^{-1} KH_2PO_4, 0.6 mol L^{-1} $Na_2HPO_4 \cdot 2H_2O$) to remove excess medium. The pellet obtained after centrifugation was immediately used for further studies.

2.9 Yeast cell encapsulation by different shape of NPs

Yeast was encapsulated with various shapes of silica NPs using the electrostatic-assisted assembly. First, to change the charge of silica NPs to a positive charge, 1 mg NPs were dispersed in 1 mL of Ethanol and 40 ul of TMSD was added and the reaction mixture was shaken for 2 h. After reaction, amino-modified NPs (positively charged) were centrifuged and washed for further use. Next, 1 mg of yeast (4 x 10^8 cells mL^{-1}), obtained after centrifugation of a 5 mL culture, was mixed with 0.3 mg of silica NPs suspension in PBS for 5 min at room temperature. Subsequently, the encapsulated yeast was collected by centrifugation, washed twice in PBS (2000 rpm, 3 minutes), and resuspended in 1 mL of PBS.

2.10 Determination of cell-growth characteristics in different media

Equal number of Yeast (4 x 10^9 cells mL^{-1}) and Yeast@2D-SiNT (4 x 10^9 cells mL^{-1}) were incubated in 2 mL of YPD medium at 30 °C. The optical absorbance at 600 nm (OD_{600}) values of 10 times diluted cultures were monitored using a photoelectric colorimeter (DiluPhotometer) at different times.

To investigate cell growth on solid YPD media, equal number of Yeast and Yeast@2D-SiNT were distributed on the solid YPD medium dishes. After incubation at 30 °C for different times, colony counting was carried out.

2.11 Cell viability estimations

Yeast (4 x 10^9 cells mL^{-1}) and Yeast@2D-SiNT (4 x 10^9 cells mL^{-1}) suspensions were dispensed in 5 mL of PBS. At different times (0, 3, 7 days), the cells were stained with fluorescein diacetate (FDA), propidium iodide (PI), and the viability of the cells was determined using flow cytometry analysis (Beckman Coulter (CytoFLEX)).

2.12 Tolerance of alcohol

Yeast (4 x 10^9 cells mL^{-1}) and Yeast@2D-SiNT (4 x 10^9 cells mL^{-1}) suspensions were dispensed in 5 mL of PBS. 2-propanol (10%, 20%, 30% v/v) were added to the cell. After incubation for 30 min, the cells were stained with fluorescein diacetate (FDA), propidium iodide (PI), and the viability of the cells was determined using flow cytometry analysis (Beckman Coulter (CytoFLEX)).

2.13 Tolerance of temperature

Yeast (4 x 10^9 cells mL^{-1}) and Yeast@2D-SiNT (4 x 10^9 cells mL^{-1}) suspensions were dispensed in 5 mL of PBS. The cells were heated in a heating shaker at (50 ℃, 60 ℃, 70 ℃) for 30 min. The cells were stained with fluorescein diacetate (FDA), propidium iodide (PI), and the viability of the cells was determined using flow cytometry analysis (Beckman Coulter (CytoFLEX)).

2.14 Tolerance of physical stress

Yeast (4 x 10^9 cells mL^{-1}) and Yeast@2D-SiNT (4 x 10^9 cells mL^{-1}) suspensions were dispensed in 5 mL of PBS. The cells were physically stimulated with a magnetic bar at 1400 RPM in a 5 ml vial for (24 h, 48 h, 72 h.) and otherwise treated sonication (40 kHz, 300W) for (30 min, 60min, 90min.) at 4 °C. The cells were stained with fluorescein diacetate (FDA), propidium iodide (PI), and the viability of the cells was determined using flow cytometry analysis (Beckman Coulter (CytoFLEX)).

2.15 Tolerance of ion strength

Yeast (4 x 10^9 cells mL^{-1}) and Yeast@2D-SiNT (4 x 10^9 cells mL^{-1}) suspensions were dispensed in 5 mL of PBS. The cells were incubated in PBS (3X, 5X, 10X) for 2 h. The cells were stained with fluorescein diacetate (FDA), propidium iodide (PI), and the viability of the cells was determined using flow cytometry analysis (Beckman Coulter (CytoFLEX)).

2.16 Tolerance of pH

Yeast (4 x 10^9 cells mL^{-1}) and Yeast@2D-SiNT (4 x 10^9 cells mL^{-1}) suspensions were dispensed in 5 mL of PBS. The cells were incubated in PBS (pH 10, 8, 6, 4, 2) for 2 h. The cells were stained with fluorescein diacetate (FDA), propidium iodide (PI), and the viability of the cells was determined using flow cytometry analysis (Beckman Coulter (CytoFLEX)).

2.17 Tolerance toward ROS

Yeast (4 x 10^9 cells mL^{-1}) and Yeast@2D-SiNT (4 x 10^9 cells mL^{-1}) suspensions were dispensed in 5 mL of PBS. The cells were incubated in H_2O_2 (3mM, 5mM, 10mM) for 2 h. The cells were stained with fluorescein diacetate (FDA), propidium iodide (PI), and the viability of the cells was determined using flow cytometry analysis (Beckman Coulter (CytoFLEX)).

2.18 Tolerance toward UV exposure

Yeast (4 x 10^9 cells mL^{-1}) and Yeast@2D-SiNT (4 x 10^9 cells mL^{-1}) suspensions were dispensed in 5 mL of PBS. The cells were placed in a dark chamber equipped with compact UV lamps (4 W lamps, 254 nm) for (2 h, 4 h, 6 h.) The cells were stained with fluorescein diacetate (FDA), propidium iodide (PI), and the viability of the cells was determined using flow cytometry analysis (Beckman Coulter (CytoFLEX)).

2.19 Cytoprotection test to Lyticase (Arthrobacter luteus)

Yeast (4 x 10^9 cells mL^{-1}) and Yeast@2D-SiNT (4 x 10^9 cells mL^{-1}) suspensions were dispensed in 5 mL of PBS. The Lyticase (10μL, 20μL, 30μL) (Arthrobacter luteus, 20000 unit/mL) were added to the

cell and incubation for 2 h. The cells were stained with fluorescein diacetate (FDA), propidium iodide (PI), and the viability of the cells was determined using flow cytometry analysis (Beckman Coulter (CytoFLEX)).

2.20 Cytoprotection test to Toxic NPs

Yeast (4 x 10^9 cells mL^{-1}) and Yeast@2D-SiNT (4 x 10^9 cells mL^{-1}) suspensions were dispensed in 5 mL of PBS. The silver nanoparticles (20μg, 40μg, 60μg) were added to the cell and incubation for 2 h. The cells were stained with fluorescein diacetate (FDA), propidium iodide (PI), and the viability of the cells was determined using flow cytometry analysis (Beckman Coulter (CytoFLEX)).

2.21 CLSM studies of Yeast

A solution of yeast (4 x 10^8 cells mL^{-1}, 1 mg mL^{-1} in DI), fluorescein diacetate (FDA, 10 μL, 5 mg mL^{-1} in acetone), and propidium iodide (PI, 50 μL, 2 mg mL^{-1} in PBS) was vortexed for 1 h at room temperature to efficiently adsorb dye molecules inside yeast. Excess dye was removed through multiple centrifugation and washing steps with DI and sample was prepared for CLSM imaging.

2.22 CLSM studies of Yeast@2D-SiNTs

A solution of Yeast (4 x 10^8 cells mL^{-1}, 1 mg mL^{-1} in DI) and 1,1'-Dioctadecyl-3,3,3',3'-Tetramethylindodicarbocyanine, 4-Chlorobenzenesulfonate Salt (DiD Solid, 10 μL, 1 mM in PBS) was vortexed for 20 min at room temperature. Excess dye was removed through multiple centrifugation and washing steps with DI water. A solution of 2D-SiNTs (0.3 mg mL^{-1} in DI) and Fluorescein isothiocyanate (FITC, 10 μL, 5 mg mL^{-1} in a mixture of DMSO and water) was vortexed for 1 h at room temperature, and excess dye was removed by multiple centrifugation and washing steps with DI water. The resulting fluorescent yeast and SiNTs were mixed to synthesize Yeast@2D-SiNTs and sample was prepared form CLSM imaging.

2.23 Cell cross-section imaging with TEM

For cell cross-section imaging, Yeast@2D-SiNTs were detached from the well plate and washed with PBS solution. Over 5 × 10^5 cells were fixed for 4 hours with modified Karnovsky's fixative (2% paraformaldehyde and 2% glutaraldehyde in 0.05 M sodium cacodylate buffer, pH 7.2). After repeated washing with 0.05 M sodium cacodylate buffer (pH 7.2) at 4°C, cells were fixed with 1% osmium tetroxide in 0.05 M sodium cacodylate buffer (pH 7.2) for 2 hours and then washed with distilled water twice. Fixed cells were en bloc stained at 4°C overnight using 0.5% uranyl acetate and then dehydrated with a graded concentration series of ethanol (30%, 50%, 70%, 80%, 90%, 100%, 100%, and 100% ethanol; 10 minutes for each dehydration step). Infiltrated cells using propylene oxide and EPON resin were polymerized at 70°C for 24 hours. Various sections of the resin block were cut using an

ultramicrotome (MT-X, RMC, Tucson, AZ, USA) and stained with 2% uranyl acetate and Reynolds' lead citrate for 7 minutes, followed by transferring the section of interest onto a 300-mesh copper TEM grid.

2 .24 Cell sample preparation for SEM

The silica wafers were cut into 1x1 cm pieces and sonicated in ethanol and acetone for 5 min each to remove impurities. They were then dried at room temperature and a cell suspension (4 x 10^{10} cells mL^{-1}) was carefully dropped with a 40 µL micropipette. After the solvent was dried at room temperature, a thin layer of Pt was coated to increase the sample's conductivity. Sample SEM images were acquired using an acceleration voltage of 5 kV and an emission current of 10 µA.

2 .25 Encapsulation of polystyrene microbeads by 2D-SiNTs

Polystyrene of different sizes (500 nm and 1000 nm) were encapsulated with positively charged 2D-SiNTs using electrostatic assisted encapsulation method. One milligram of polystyrene was mixed with 0.3 mg of 2D-SiNTs suspension in DI for 5 min at room temperature. The encapsulated polystyrene was then collected by centrifugation, washed twice with PBS (2000 rpm, 3 min), and resuspended in 1 mL of PBS.

2.26 Recycling test of bio-reduction using Yeast@2D-SiNTs

A suspension of Yeast@2D-SiNT (4 x 10^{10} cells mL^{-1}) grown in PBS (5 mL) was gently shaken at 25°C for 30 minutes. Methyl benzoylformate (0.03 mmol) was combined with 2-propanol (80 µL), and this mixture was added to the yeast suspension and stirred for 9 h. The reaction mixture was centrifuged at 2000 RPM for 5 min to separate and extract the supernatant. The remaining catalyst pellet was washed twice with PBS, and used for 5 cycles.

2.27 Gating strategy for flow cytometry experiments

Using flow cytometry analysis (Beckman Coulter (CytoFLEX)), the sample flow rate was 10uL/min, 20,000 to 40,000 Events were collected, and the last 10,000 Events collected were used. Live cells were separated by green, fluorescein diacetate (FDA), and dead cells were separated by red, propidium iodide (PI). Afterwards, using CytExpert software, the y-axis was set to B525-FITC and the x-axis was set to R660-APC to gate the number of cells.

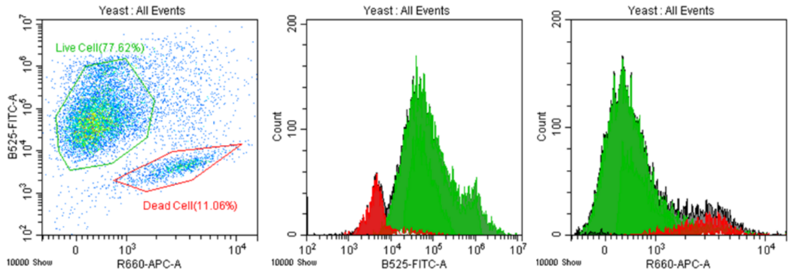

Supplementary Figures

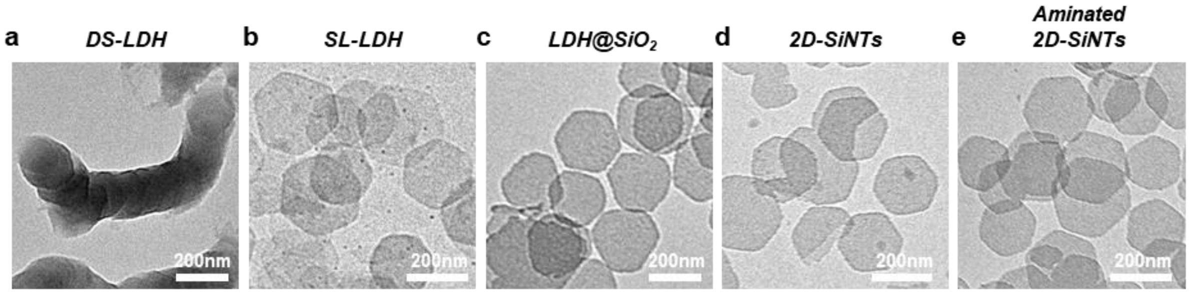

f

Materials	Ni (wt%)	Co (wt%)	Si (wt%)
DS-LDH	68.54	31.46	-
SL-LDH	67.63	32.37	-
LDH@SiO_2	33.68	12.89	53.43
2D-SiNTs	0	0	100

Supplementary Fig. 1. TEM image of (a) DS-LDH, (b) SL-LDH, (c) LDH@SiO_2, (d) 2D-SiNTs, and (e) aminated 2D-SiNTs. (f) Elemental composition of different materials detected by TEM-EDS analysis.

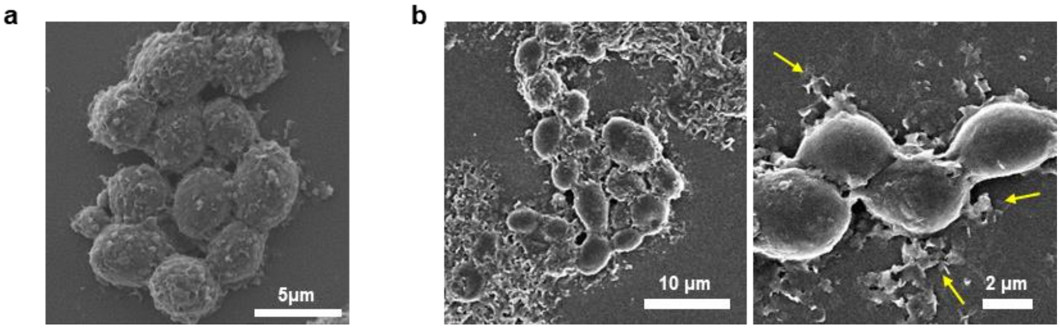

Supplementary Fig. 2. (a) SEM image of Yeast cells encapsulation using positively charged 2D-SiNTs. (b) SEM images of Yest cell encapsulation using negatively charged 2D-SiNTs; yellow arrows showing deattached 2D-SiNTs from cell surface due to electrostatic repulsion.

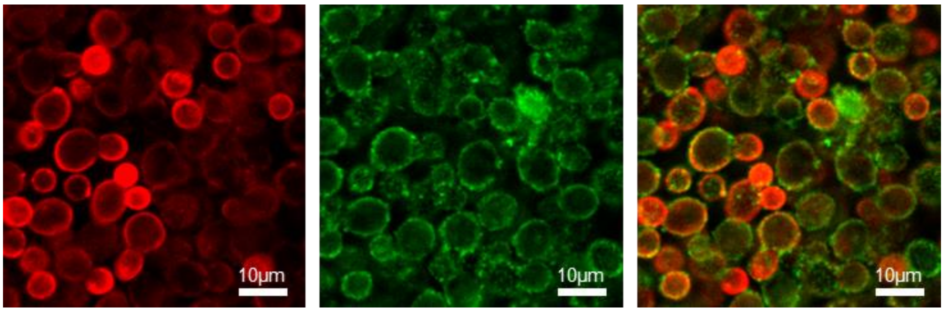

Supplementary Fig. 3. Large area CLSM fluorescence images of Yeast@2D-SiNTs in red, green and merged channels. Yeast is stained with DiD Solid (red), and 2D-SiNTs are stained with FITC (green).

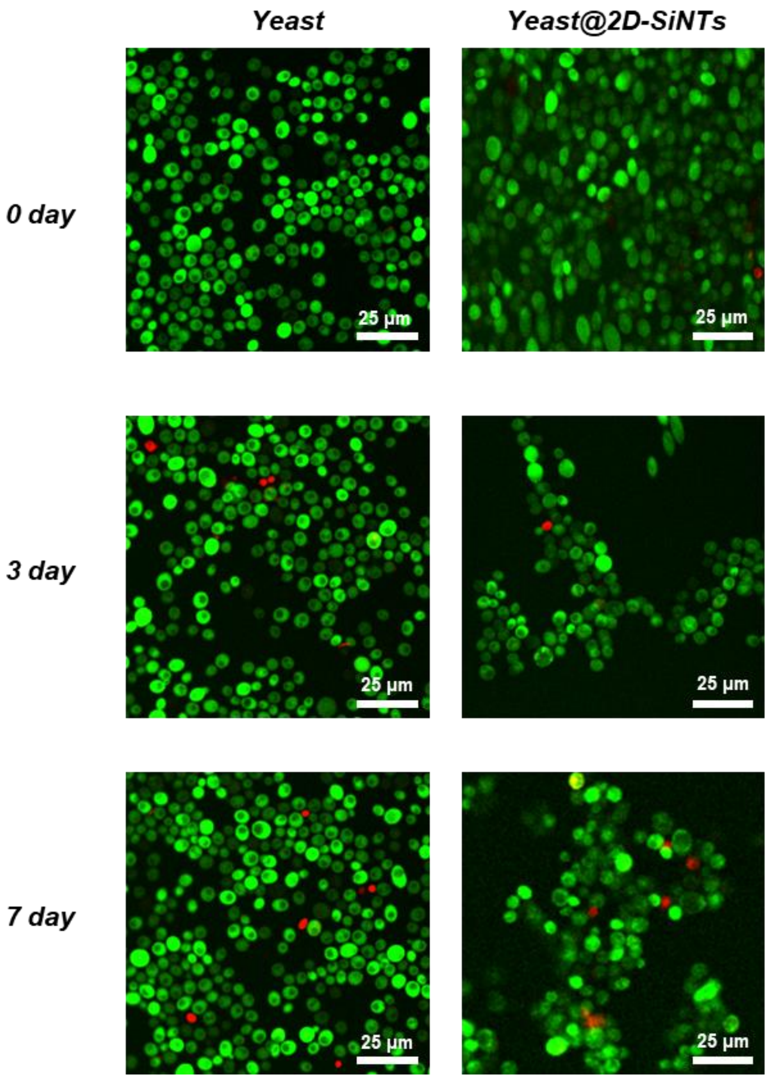

Supplementary Fig. 4. Fluorescence images for live/dead cell assay of yeast and Yeast@2D-SiNTs. Cells were stained with FDA (green: live cell), and PI (red: dead cell).

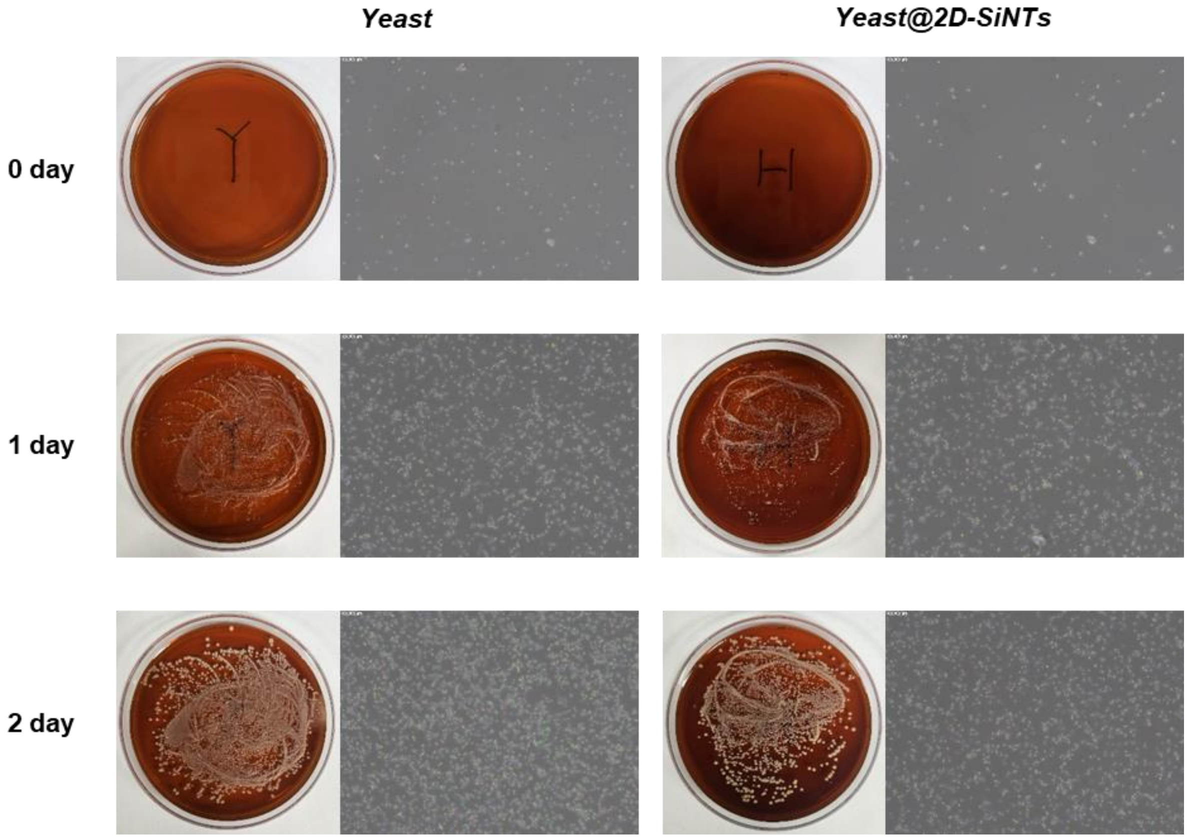

Supplementary Fig. 5. Monitoring time-dependent cell growth on solid YPD agar plates. Images were captured at intervals of 0, 1, and 2 days on YPD agar petri-dish (left). The development of yeast growth was also observed under an optical microscope (right).

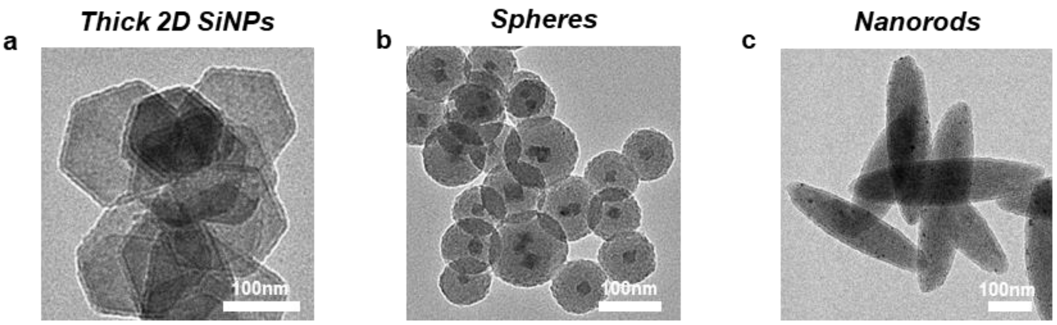

Supplementary Fig. 6. TEM images of different silica nanoparticle shapes used for assembly on cell. (a) thick 2D-silica nanosheets; (b) silica nanospheres; (c) silica nanorods.

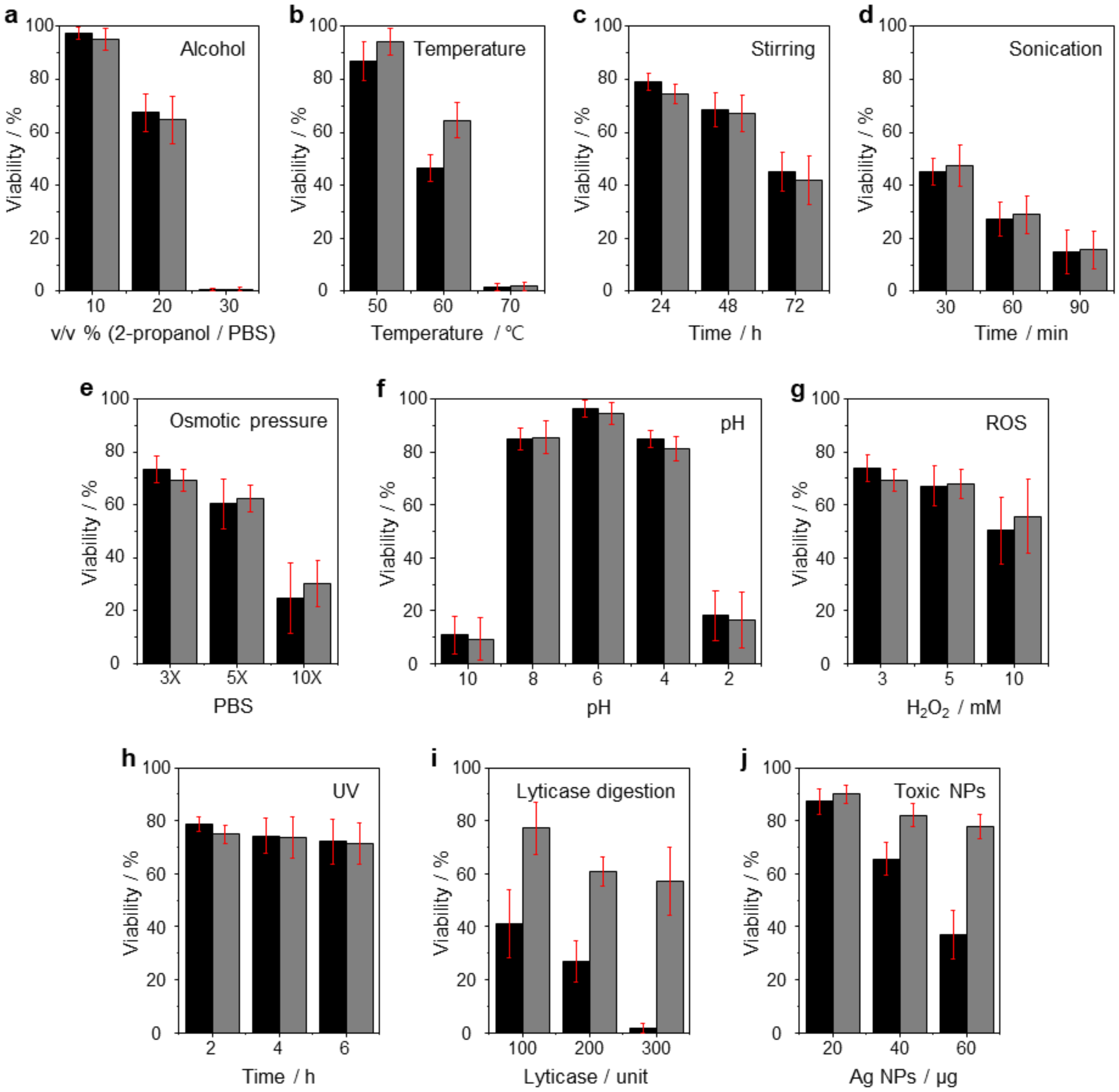

Supplementary Fig. 7. Resistance of Yeast@2D-SiNTs to endogenous and exogenous stimuli. Viability of yeast (black) and Yeast@2D-SiNTs (grey) after treatment with different alcohol (2-propanol, 30 min) concentrations (a); different temperatures for 30 min (b); physical stresses (stirring 1400 RPM, sonication (40 kHz 300 W), osmotic pressures (PBS, 2 h) (c-e); different pH for 2h (f); different ROS (H_2O_2) concentrations for 2 h (g); UV (254 nm 4W) for 2 h (h); different amounts of Lyticase enzyme for 2 h (i); toxic NPs (Ag NPs 20 nm) for 2 h (j). Error bars in the inset plot represent standard deviation of the mean obtained by 3 independent experiments.

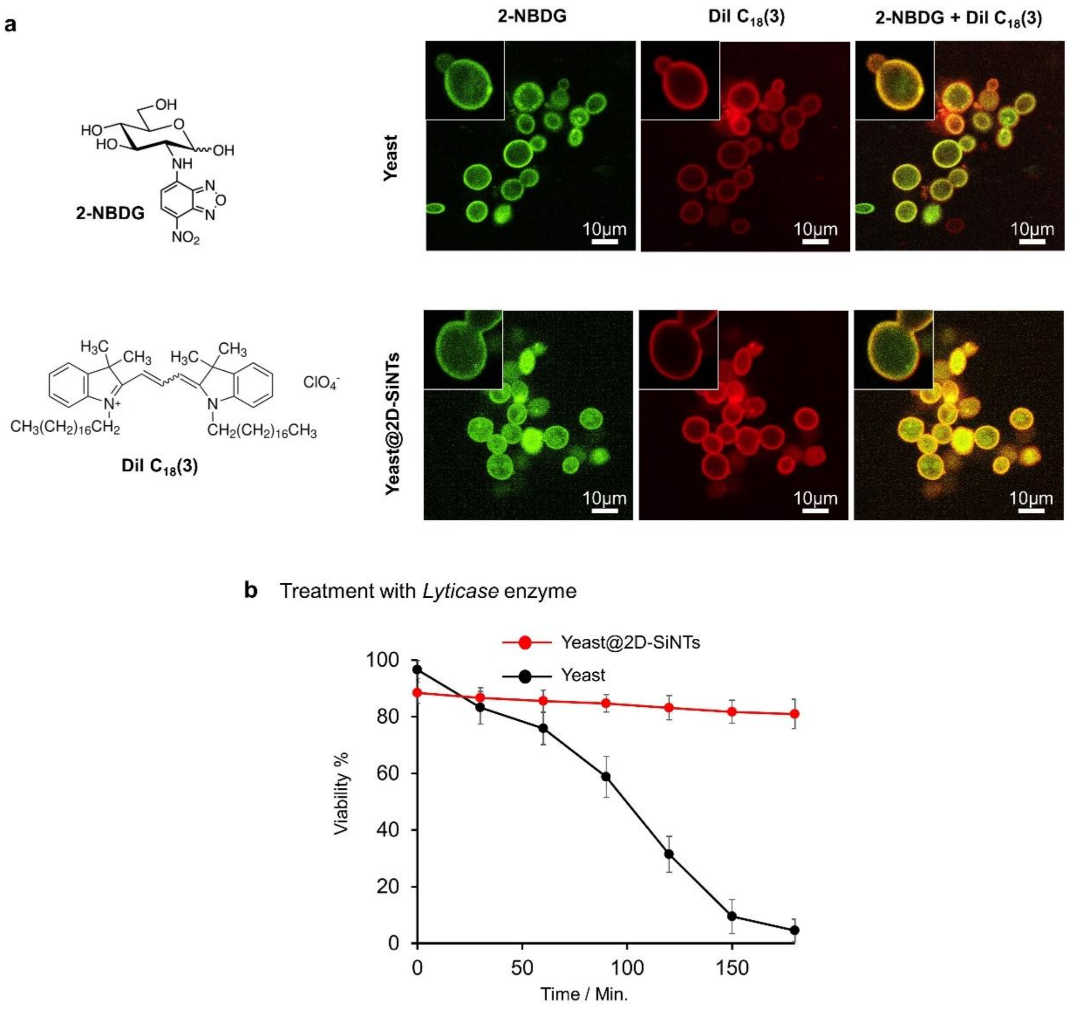

Supplementary Fig. 8. **Diffusive characteristics of Yeast@2D-SiNTs.** (a) CLSM based fluorescence images (left to right: green, red, and merged) showing facile uptake of 2-NBDG, a fluorescent tracer (green) in contrast to the membrane impermeable Dil $C_{18}(3)$ (red), in both the cases (Yeast and Yeast@2D-SiNTs). (b) Cell viabilities of Yeast and Yeast@2D-SiNTs after treating with *Lyticase* enzyme for different times. Error bars in the inset plot represent standard deviation of the mean obtained by 3 independent experiments.

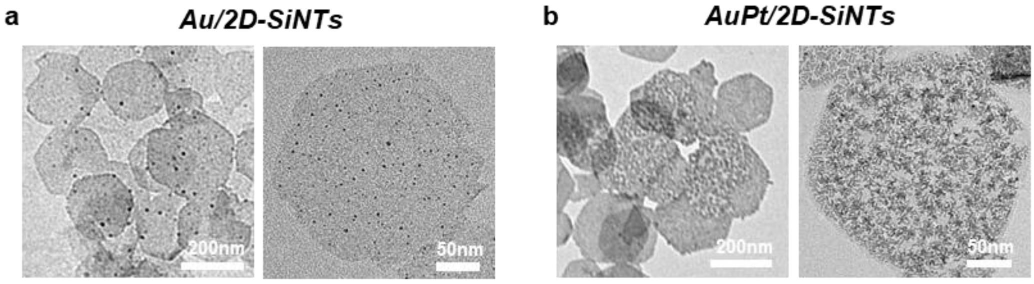

c

Materials	Au (wt%)	Pt (wt%)
Au@2D-SiNTs	8.63	-
AuPt@2D-SiNTs	6.44	21.64

Supplementary Fig. 9. TEM images of (a) Au/2D-SiNTs and (b) AuPt/2D-SiNTs. (c) Elemental composition (Au and Pt) detected by ICP-AES.

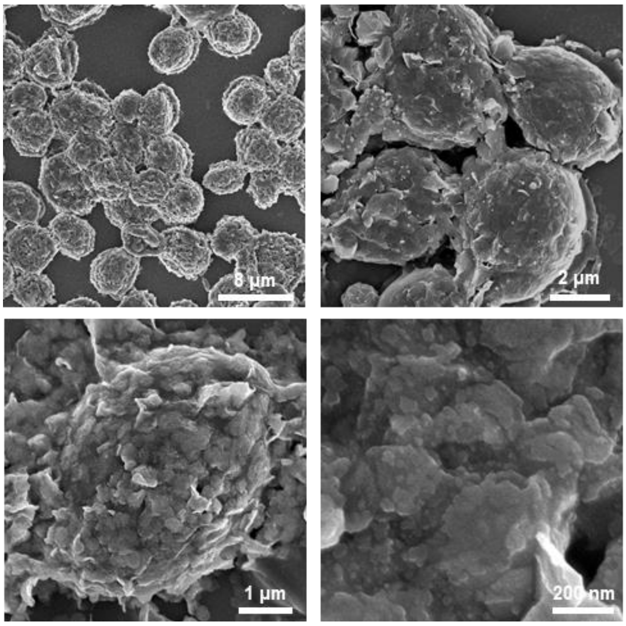

Supplementary Fig. 10. SEM images of AuPt/2D-SiNTs.

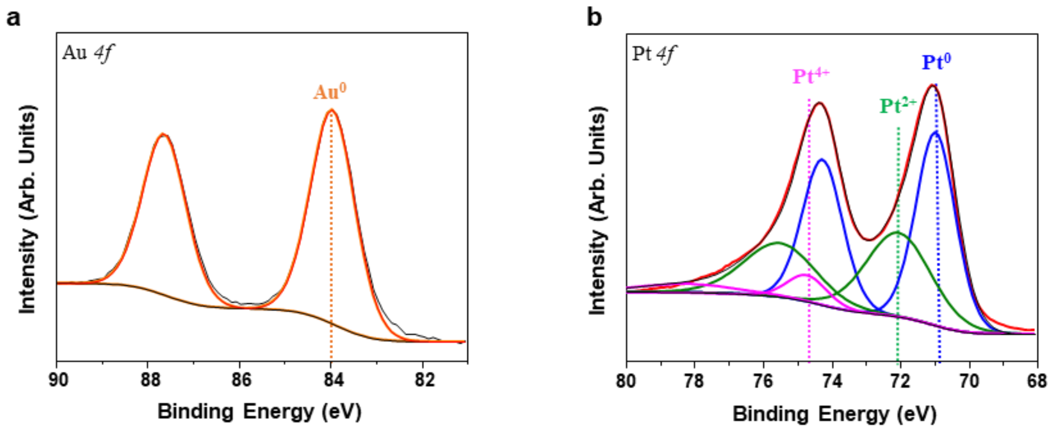

Supplementary Fig. 11. XPS plots of AuPt/2D-SiNTs showing the chemical states of Au *4f* (a) and Pt *4f* (b).

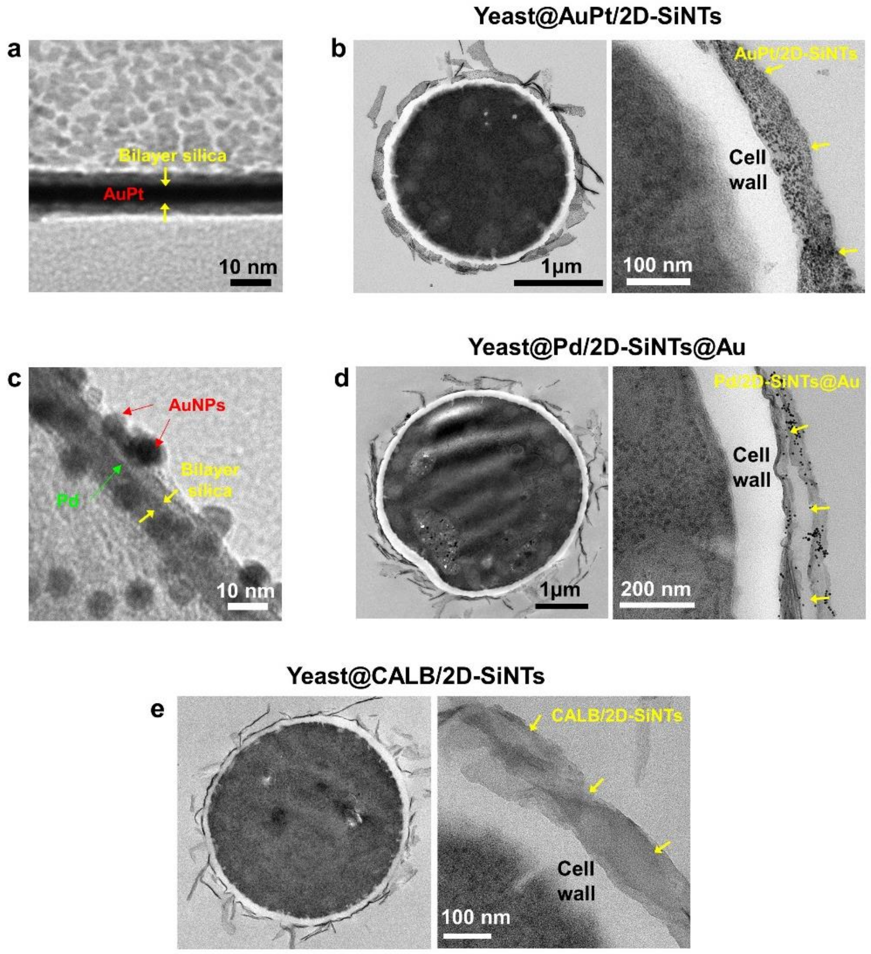

Supplementary Fig. 12. TEM images showing detailed characterization of different components of Yeast nanobiohybrid catalysts. (a,c) TEM images of AuPt/2D-SiNT and Pd/2D-SiNT@Au single-sheet in standing orientation. (b,d,e) Bio-TEM images of different cells in ultra-microtomed samples.

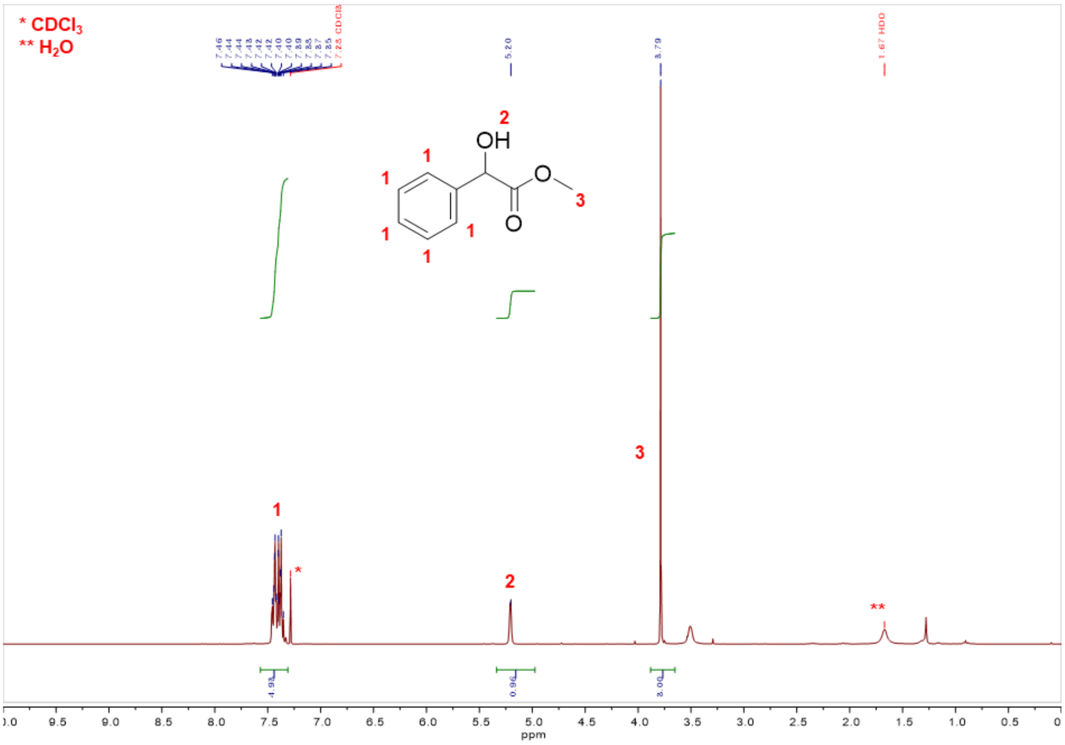

Supplementary Fig. S13. ^{1}H NMR spectrum (500 MHz) of the alcohol after reduction of methyl mandelate using Yeast@AuPt/2D-SiNTs recorded in $CDCl_3$.

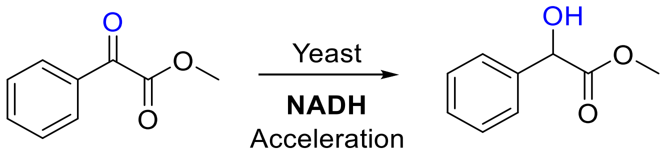

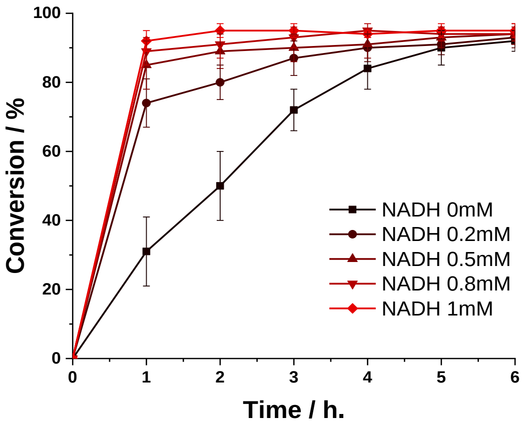

Supplementary Fig. 14. The rate of methyl benzoyl formate reduction after adding diiferent concentration of NADH in to reaction solution. Error bars in the plots represent standard deviation of the mean obtained by 3 independent experiments.

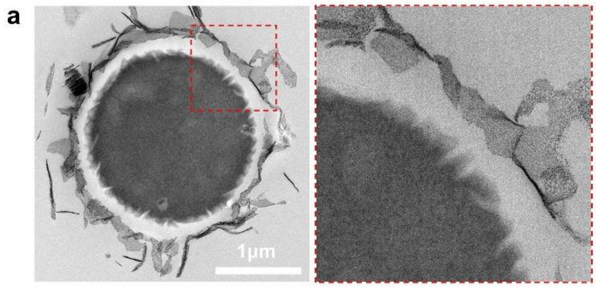

b

ICP-AES analysis

Yeast@AuPt /2D-SiNTs	Before Catalysis		After Catalysis	
	Au (wt%)	Pt (wt%)	Au (wt%)	Pt (wt%)
**Supernatant**	0.085 (±0.072)	0.101 (±0.097)	0.040 (±0.143)	0.064 (±0.171)
**Pellet**	8.177 (±1.028)	19.588 (±2.563)	8.450 (±1.824)	20.019 (±2.824)

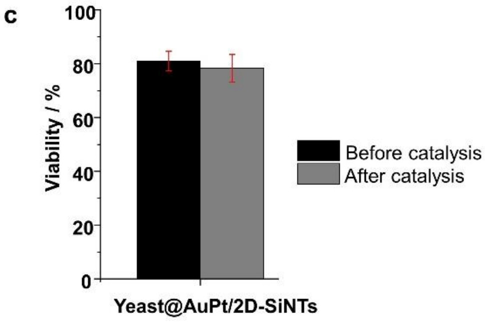

Supplementary Fig. 15. **Stability test of Yeast@AuPt/2D-SiNTs.** (a) Bio-TEM images of ultra-microtomed samples showing intact morphology of *tiles* after using in the catalysis reaction; (b) ICP-AES analysis to confirm leaching of metals; (c) Cell viability before and after using the catalyst in the reaction. Error bars in the plots represent standard deviation of the mean obtained by 3 independent experiments.

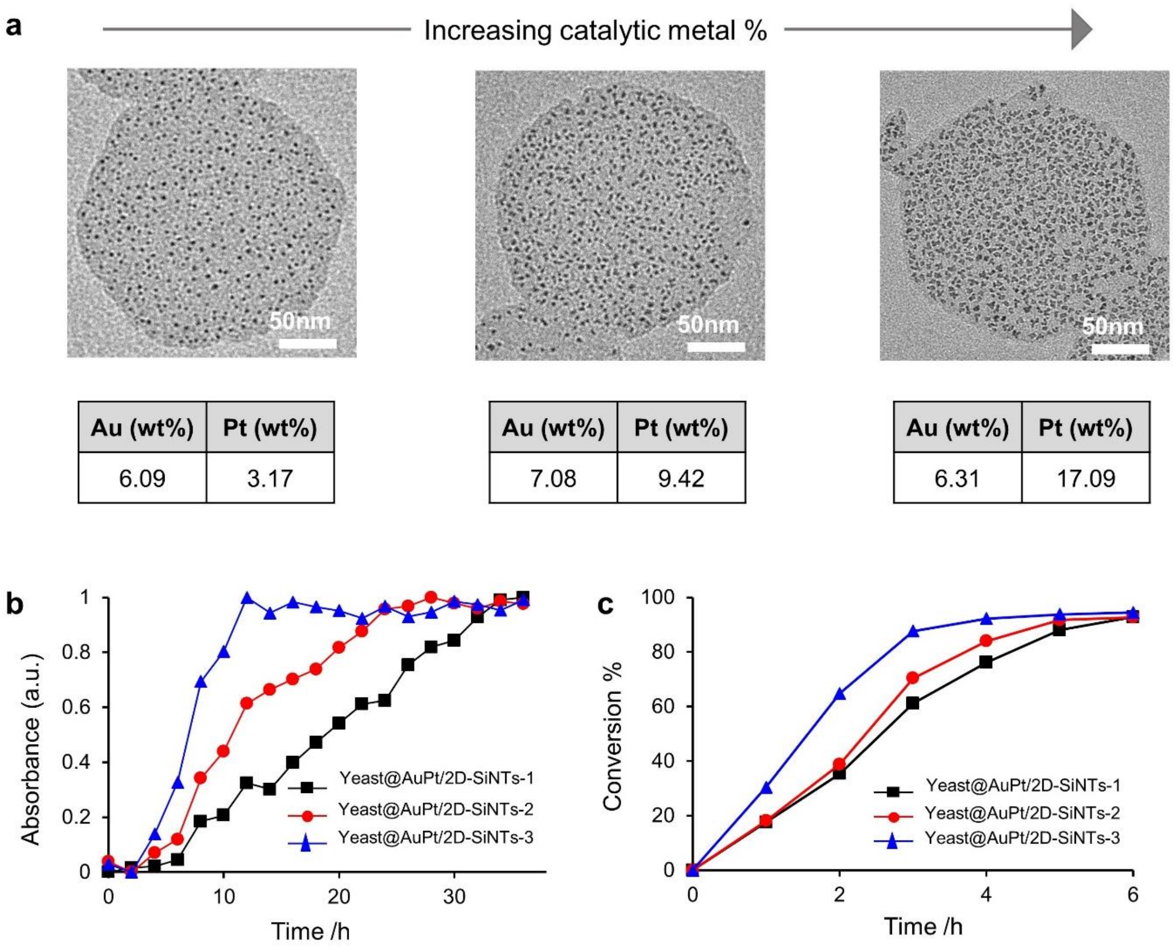

Supplementary Fig. 16. Catalytic reaction test of Yeast@AuPt/2D-SiNTs with variable amounts of metals. (a) TEM images and EDS-based metal wt% data of different AuPt/2D-SiNTs; (b) NADH generation rates of different reactions using catalysts (1-3: increasing Pt amounts) based on the increase in absorbance at 360 nm in UV-vis spectrophotometry; (c) Corresponding reaction kinetics of methyl benzoylformate bio-reduction in the presence of NAD^+, as measured by ^1H NMR.

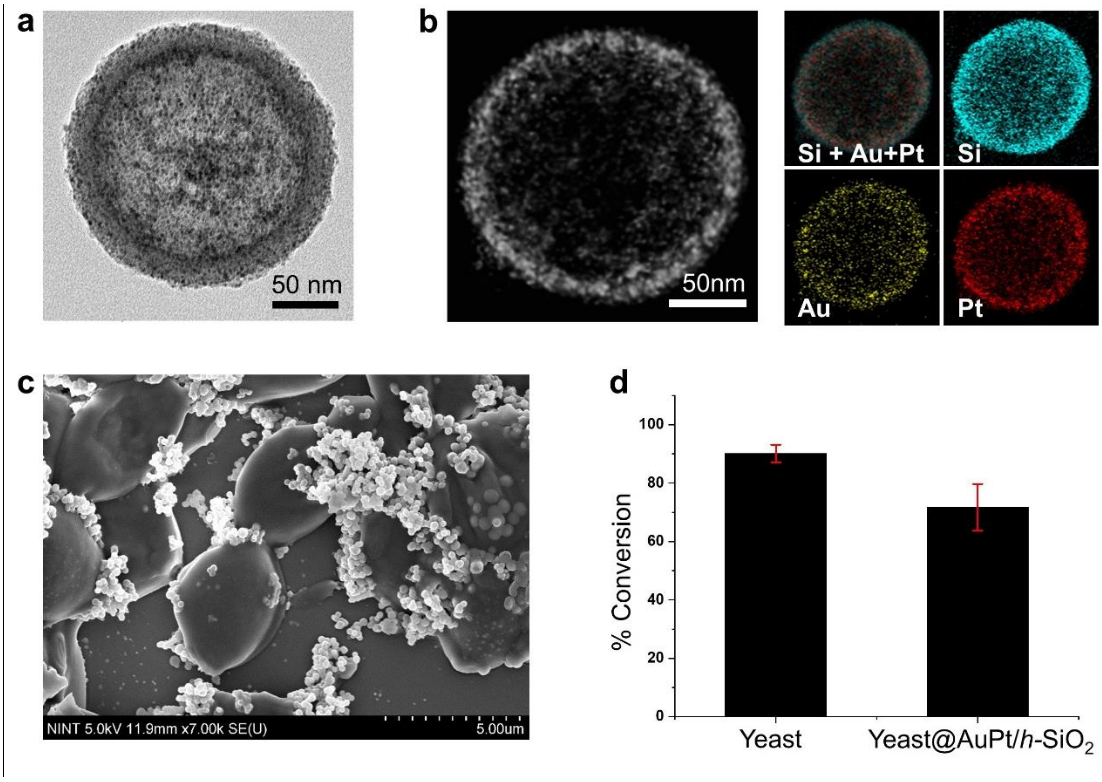

Supplementary Fig. 17. Characterization and catalysis test of Yeast@AuPt/*h*-SiO_2 as control study. (a-b) TEM, HAADF-STEM and EDS-based elemental mapping of AuPt/*h*-SiO_2 NPs; (c) SEM image of Yeast@AuPt/*h*-SiO_2 showing random clustering of AuPt/*h*-SiO_2 on cell surface; (d) Reduction of methyl benzoylformate to the methyl 2-hydroxy-2-phyenyl acetate in the presence of exogenous β-NAD^+ using Yeast@AuPt/*h*-SiO_2; Error bars in the plots represent standard deviation of the mean obtained by 3 independent experiments.

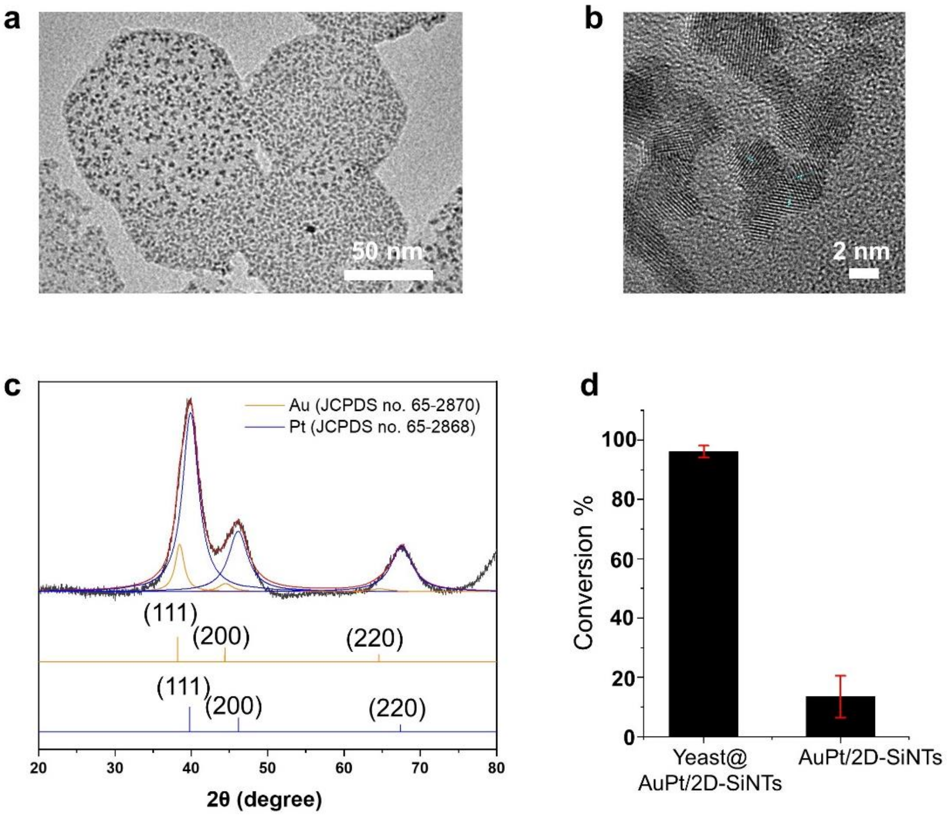

Supplementary Fig. 18. **Characterization and catalytic reaction test of AuPt/2D-SiNTs;** (a-b) TEM and HRTEM images of AuPt/2D-SiNTs, showing Au-seed mediated dendritic growth of Pt; (c) XRD data of AuPt/2D-SiNTs showing existence of Au and Pt simple mixture. (d) Conversion yield of methyl benzoylformate bio-reduction in the presence of NAD^+ using AuPt/2D-SiNTs (without Yeast), as measured by ^1H NMR. Error bars in the plots represent standard deviation of the mean obtained by 3 independent experiments.

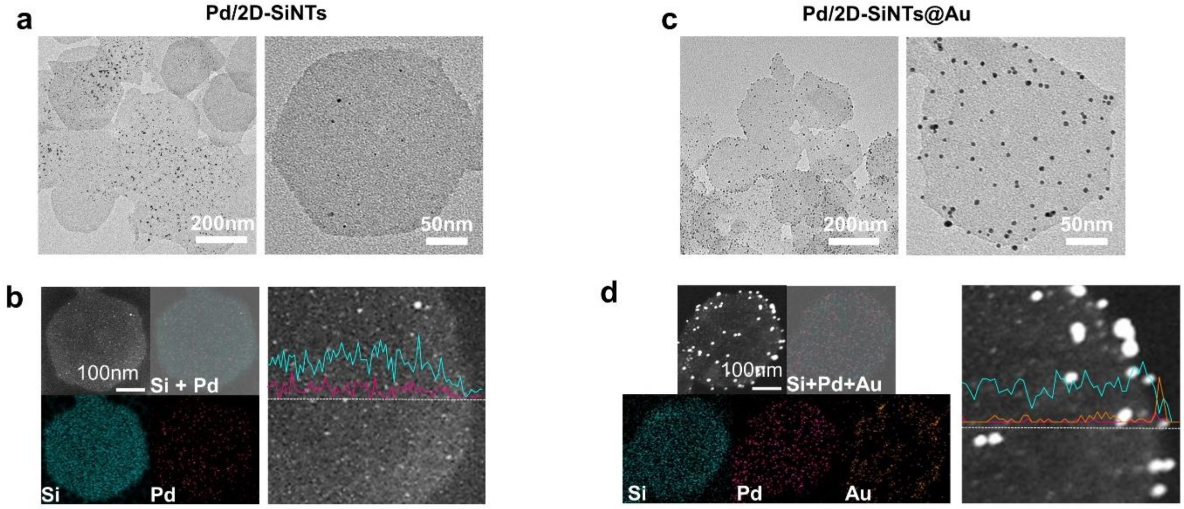

e

Materials	Pd (wt%)	Au (wt%)
Pd/2D-SiNTs	2.43	-
Pd/2D-SiNTs@Au	2.59	5.66

Supplementary Fig. 19. (a-b) TEM and STEM-EDS based elemental mapping and line-profiling of Pd/2D-SiNTs; (c-d) TEM and STEM-EDS based elemental mapping and line-profiling of Pd/2D-SiNT@Au; (e) Elemental composition (Au and Pd) detected by ICP-AES.

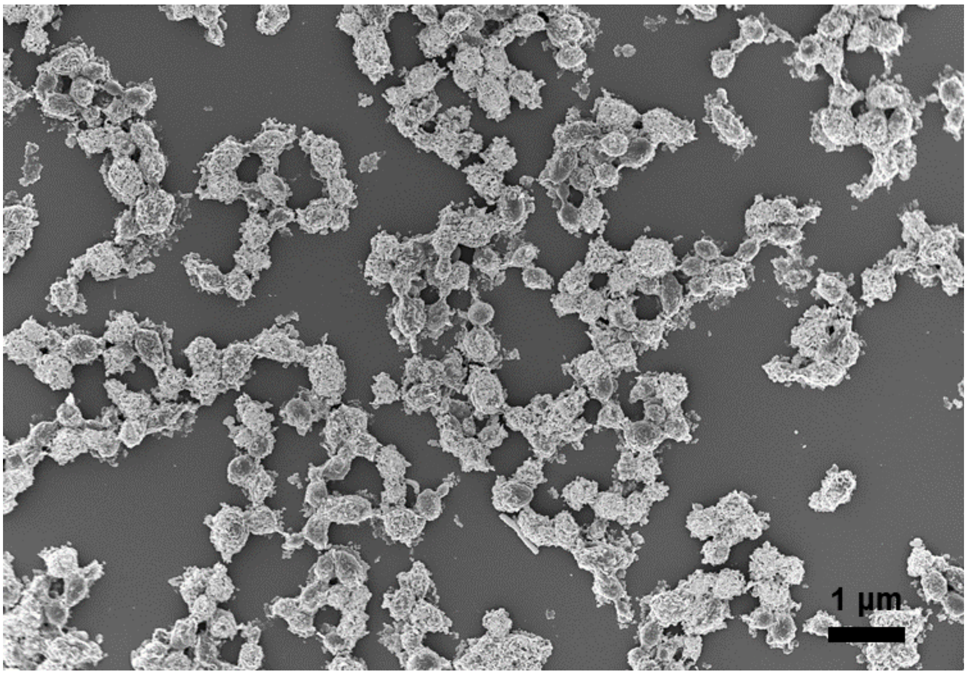

Supplementary Fig. 20. SEM image of Yeast@Pd/2D-SiNTs@Au.

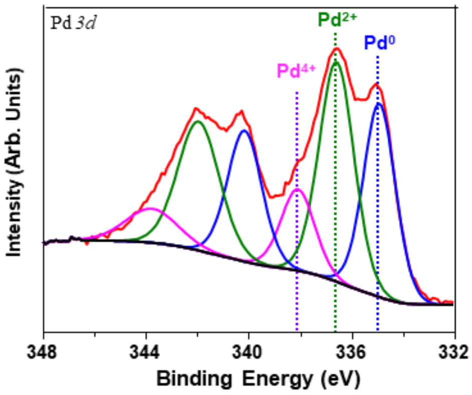

Supplementary Fig. 21. Pd 3d XPS spectra for Pd@2D-SiNTs.

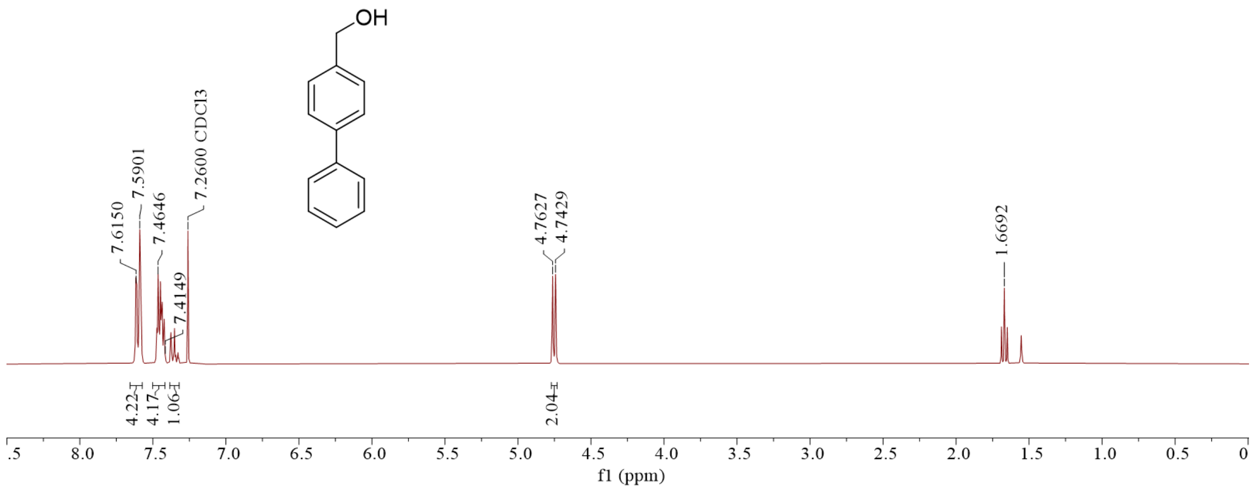

Supplementary Fig. 22. ^{1}H NMR spectrum (500 MHz) of Biphenyl methanol resulted from the plasmon-induced Pd catalyzed Suzuki coupling and yeast reduction using Yeast@Pd/2D-SiNT@Au, recorded in $CDCl_3$.

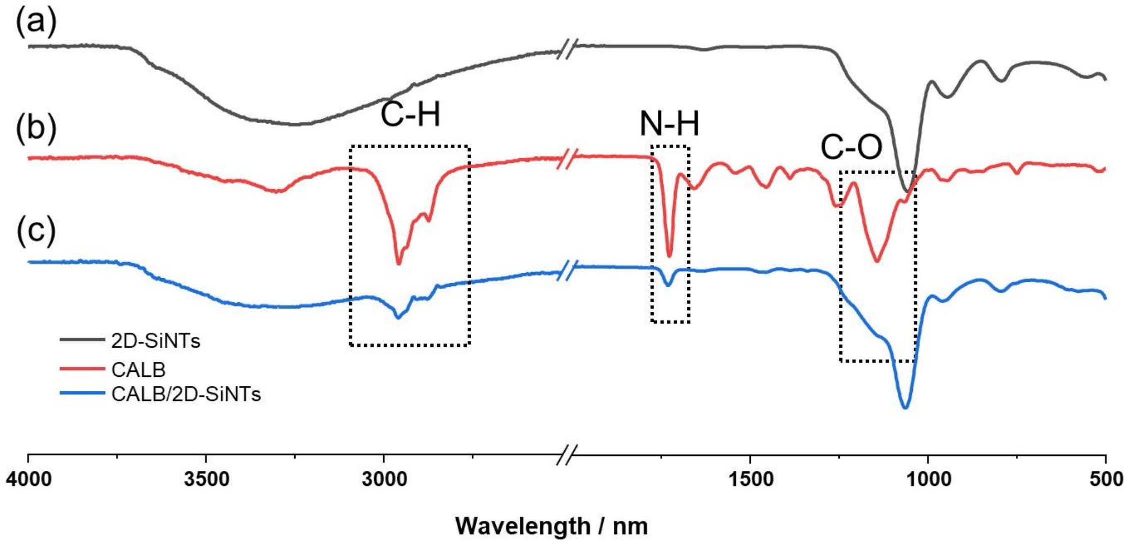

Supplementary Fig. 23. FTIR spectra of 2D-SiNTs (a); pure CALB enzyme (b); CALB/2D-SiNTs (c).

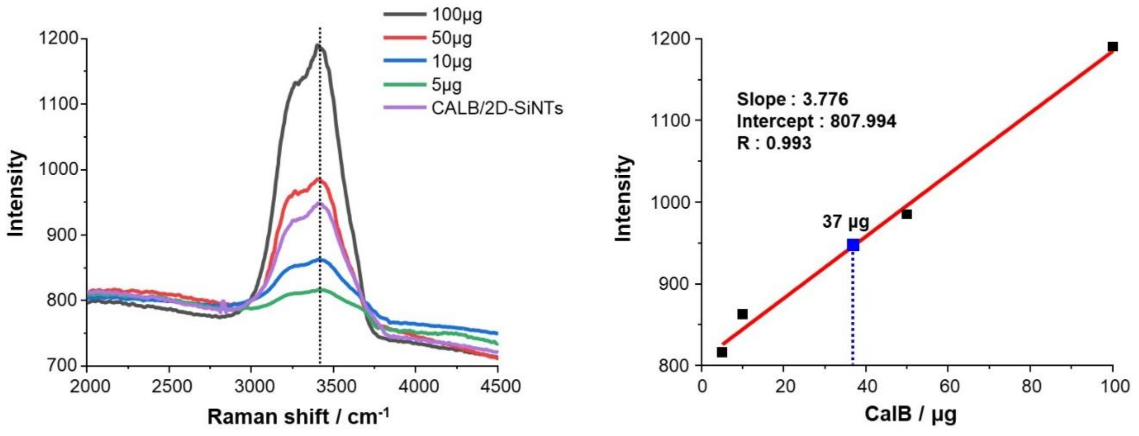

Supplementary Fig. 24. Raman spectra of CALB versus weight for calibration. Calibration curve of intensity versus weight at 3395 cm^{-1} estimating the loading of CALB in CALB@2D-SiNTs.

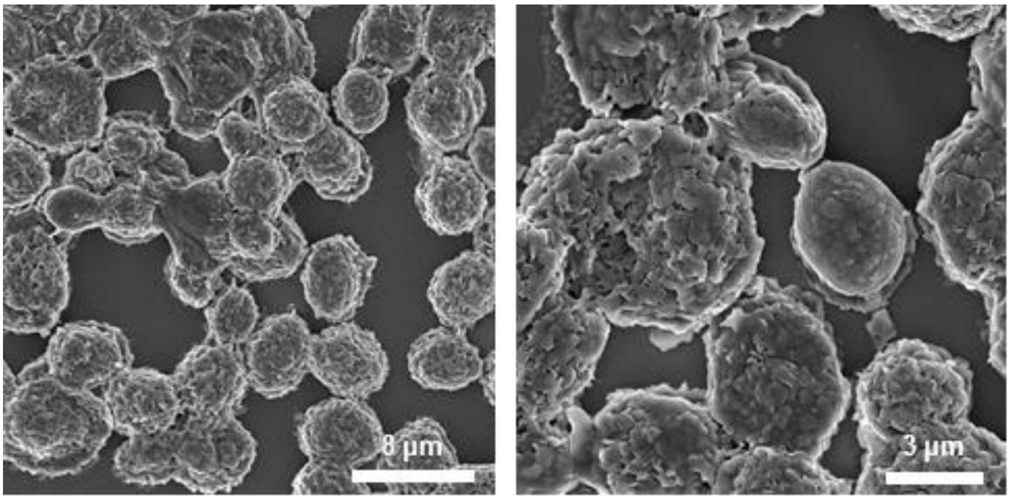

Supplementary Fig. 25. SEM image of Yeast@CALB/2D-SiNTs

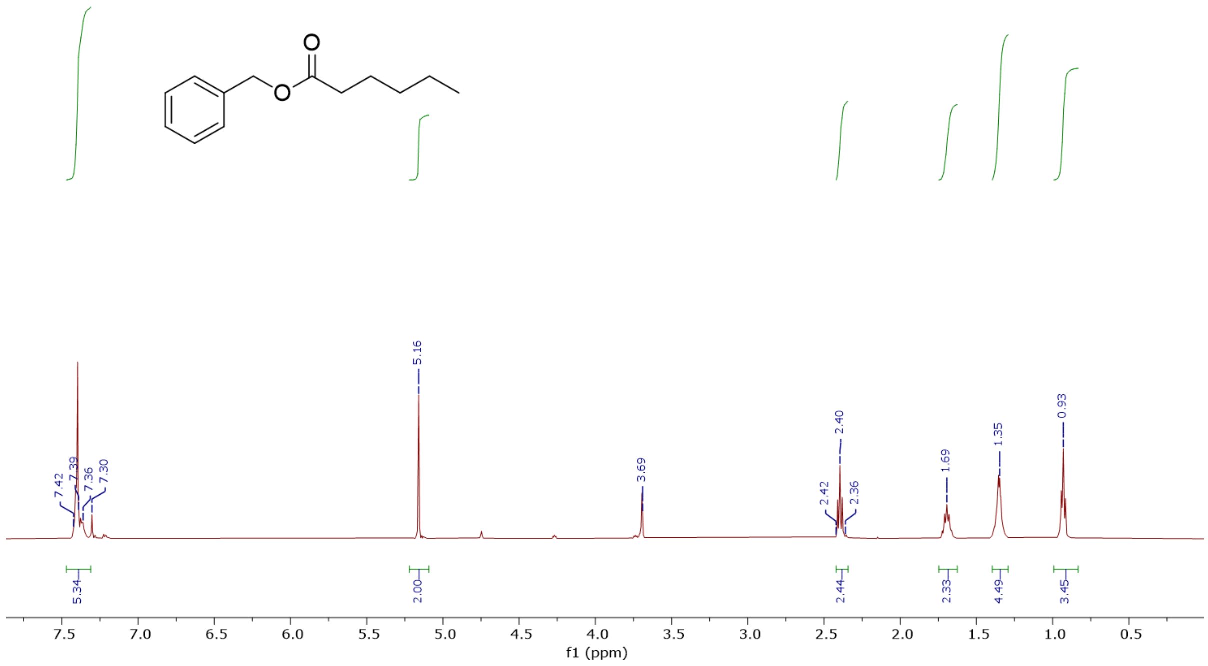

Supplementary Fig. 26. ^{1}H NMR spectrum (500 MHz) of Benzyl hexanoate after the yeast reduction and CALB catalyzed esterification using Yeast@CALB/2D-SiNTs, recorded in $CDCl_3$.

Supplementary References

(1) Ida, S., Shiga, D., Koinuma, M., Matsumoto, Y. Synthesis of hexagonal nickel hydroxide nanosheets by exfoliation of layered nickel hydroxide intercalated with dodecyl sulfate ions. *J. Am. Chem. Soc.* **130**, 14038–14039 (2008).

(2) Jang, S. W., Dutta, S., Kumar, A., Kim, S. M., You, Y.-W., Lee, I. S. Silica-enveloped 2D-sheet-to-nanocrystals conversion for resilient catalytic dry reforming of methane. *Small* **17**, 2102851 (2021).

(3) Koo, J. H., Kumar, A., Lee, S. H., Jin, X., Jeong, H., Kim, J., Lee, I. S. Pore-Engineered Silica Nanoreactors for Chemical Interaction-Guided Confined Synthesis of Porous Platinum Nanodendrites. *Chem. Mater.* **30**, 3010-3018 (2018).

(4) Bastús, N. G., Merkoçi, F., Piella, J., Puntes, V. Synthesis of Highly Monodisperse Citrate-Stabilized Silver Nanoparticles of up to 200 nm: Kinetic Control and Catalytic Properties. *Chem. Mater.* **26**, 2836–2846 (2014).
