# Supplementary material for: Ultrathin silica-tiling on living cells for chemobiotic catalysis
Source: Nat Commun. 2024 Jul 10;15:5773. doi: 10.1038/s41467-024-50255-7 (PMC11233561; doi:10.1038/s41467-024-50255-7)
Supplement: Supplementary file 3 — Description of Additional Supplementary Files [file 41467_2024_50255_MOESM3_ESM.pdf]

Description of Additional Supplementary Files

Supplementary Movie 1 – CLSM-based 3D Z-scanning of Yeast@2D-SiNT

Supplementary Movie 2 – Real time CLSM-based video on the cell division process of Yeast@2D-SiNTs
